# Development of an improved medium for the preservation of human spermatozoa

**DOI:** 10.1007/s10815-025-03525-2

**Published:** 2025-05-30

**Authors:** Alena J. Hungerford, Natasha Harrison, Hassan W. Bakos, Robert J. Aitken

**Affiliations:** 1 School of Environmental and Life Sciences, College of Engineering, Science and Environmental Science, University of Newcastle, Callaghan, NSW 2308 Australia; 2 Memphasys Ltd., Sydney, NSW 2140 Australia; 3 Hunter Medical Research Institute, New Lambton Heights, NSW 2305 Australia

**Keywords:** Cryopreservation, Reactive oxygen species, Spermatozoa, *Myo-*inositol, Vitamin C, EDTA

## Abstract

**Purpose:**

To create a novel medium that retained human sperm quality following cryopreservation at a higher level than that seen with currently available commercial cryoprotectants.

**Methods:**

Cryopreservation was achieved via 1:1 dilution with cryoprotectant followed by slow-programmed freezing. A NaCl-free cryopreservation carrier medium based on the use of histidine as the major osmolyte was designed that was capable of sustaining human sperm motility over 6 days at ambient temperature. This medium was supplemented with ethylene glycol, glycerol, and DMSO to create the basis for a novel cryopreservation medium. Dose-dependent studies with various supplements were then conducted to optimize the effectiveness of this formulation including assessments of vitamin C, EDTA, crocin, zinc, ergothioneine, and *myo*-inositol, as well as the potential replacement of DMSO by Cyrene™. Post-thaw samples were assessed for motility, vitality, and DNA integrity and then reassessed following sperm isolation with the Felix™ System.

**Results:**

The completed cryopreservation formulation comprised 4.5% ethylene glycol, 4.5% glycerol, 1% DMSO in a carrier medium supplemented with 0.4 mM vitamin C, 1 mM EDTA, and 22 mM *myo*-inositol. Spermatozoa frozen in this medium and isolated using the Felix™ System had significantly greater total motility, progressive motility, vitality, and DNA integrity than spermatozoa frozen in a commercially available product that is widely used in infertility clinics.

**Conclusion:**

A novel cryopreservation medium has been developed in this study that represents a significant improvement over existing technologies.

**Supplementary Information:**

The online version contains supplementary material available at 10.1007/s10815-025-03525-2.

## Introduction

Human sperm quality has seen a significant global decline in recent years, reflecting the combined impact of advancing age, deteriorating environment and current lifestyle patterns on the spermatogenic process [1–4]. This has created a corresponding demand for assisted reproductive technologies (ART) including in-vitro fertilization (IVF), intra-cytoplasmic sperm injection (ICSI), and artificial insemination (AI). An important component of the ART toolset involves the use of cryopreservation to store spermatozoa in preparation for insemination at a later timepoint [5]. Typically, this procedure is used in the treatment of cancer patients, individuals requiring therapeutic interventions potentially affecting testicular function, and male partners who cannot generate a sample at the time of oocyte retrieval for a variety of reasons. The cryopreservation process itself usually involves mixing an unprocessed semen sample with a cryoprotectant followed by a progressive reduction in temperature to − 196 °C, at which point the sample is stored in a liquid nitrogen dewar [6–10]. Although the technique is effective, the underpinning technology, particularly its reliance on the use of glycerol as a cryopreservation agent, has not changed significantly since its clinical introduction in 1953 [11]. Moreover, even current cryopreservation techniques inflict considerable damage on the spermatozoa, impairing motility, vitality, and DNA integrity as a result of the physical damage inflicted by ice crystal formation, osmotic shock, and oxidative stress [12–14].

Of late, there has been an increase in studies comparing cryopreservation techniques to optimize sperm recovery from cryoinjury, and a consensus developed that either slow-programmed freezing or vitrification generates the best outcomes [14]. In contrast, no consensus has developed for the most efficient cryopreservation medium to use. Numerous studies have demonstrated that the addition of various compounds to commercial media can enhance their effectiveness with increases in motility, vitality, and DNA retention, for example, vitamin C [12], zinc sulfate [15], crocin [16], quercetin [16], and canthaxanthin [17]. Despite such studies, the impact of cryopreservation on human sperm function has remained substantially unchanged for more than half a century. Even today, sperm freezing using commercially available cryopreservation media is associated with an approximate halving of sperm motility [5] and a doubling of sperm DNA damage [18].

An optimized medium would not only need to significantly increase the retention of sperm motility, vitality, and DNA integrity post-freeze, but also ensure that reactive oxygen species (ROS) are lowered and that the plasma membrane is adequately protected [14]. If this could be achieved, the sperm cryopreservation industry would benefit as it would allow the successful banking and retrieval of spermatozoa that may previously have been irreparably damaged using traditional protocols. The purpose of this study was to systematically design and evaluate a new cryopreservation medium for human use that represents a significant improvement over existing technologies.

## Materials and methods

### Semen analysis

Human spermatozoa were obtained from the University of Newcastle reproductive research program. The mean ± SEM parameters of semen quality of the donors (*n* = 103) employed in this study were as follows: count (76.35 ± 4.95 × 10^6^/mL), vitality (75.86 ± 0.74%), total motility (58.04 ± 1.04%) and morphology (6.76 ± 0.18%). A CASA system (Hamilton Thorne, IVOS II, Beverly, MA, USA) was also used to assess progressive motility in semen (straightness of ≥ 80% and an average path velocity of ≥ 25 µm/s), generating a value of 25.65 ± 0.95%. These donors were healthy University students and staff aged 18–76 years, of unknown fertility status but selected on the basis of being free from infection and exhibiting normozoospermia according to criteria laid down by the World Health Organization [13] including a sperm concentration of ≥ 15 million/mL, total motility of ≥ 40%, morphology of ≥ 4%, and vitality of ≥ 58%. Ethical approval was secured for the use of semen samples in research from both the University of Newcastle human ethics committee and the state government (H-2013–0319 and 200621). Signed consent for research purposes was also obtained from each participant.

Samples were procured within 1-h post masturbation and collection in sterile sample containers after ≥ 48-h abstinence. Once delivered, semen analysis was conducted immediately with a focus on sperm vitality, morphology (Testsimplets Waldeck, GmbH, Münster, Germany), count and a CASA-based analysis of sperm motility (Hamilton Thorne, IVOS II, Beverly, MA, USA), as described previously [12].

### Cryopreservation and thawing

An aliquot of semen was slowly mixed 1:1 (v/v) with a cryopreservation medium that was either Quinn’s Advantage™ Sperm Freezing Medium (Cooper Surgical, Shelton, CT, USA), selected because it is the most commonly used commercial cryopreservation medium in local clinics, as a control, or a test medium. Once mixed, solutions were loaded into 0.5 mL straws and the ends heat sealed (CBS High Security Sperm Straw with Tripartite Plug, IMV, L’Aigle, France: Conception Technology, San Diego, CA, USA).

Cryopreservation was achieved by gradual cooling in liquid nitrogen by controlled slow rate freezing using the FreezeControl™ system from Cryologic (Victoria, Australia) on setting 0. Once a temperature of − 80 °C was achieved, the straws were plunged into and stored in − 196 °C liquid nitrogen. Maximum time stored in a liquid nitrogen dewar was 2 weeks.

Testing of cryopreserved samples began by removing the straws from liquid nitrogen and thawing by acclimatization to room temperature (~ 21 °C) in the laboratory over 15 min. Once at room temperature, the straw exteriors were washed with distilled water and dried, then their ends were cut and contents released into 1.5 mL Eppendorf tubes. Following thawing, samples were immediately washed twice via centrifugation at 500 g for 3 min and resuspended in Biggers, Whitten and Whittingham (BWW) medium [19] to avoid unnecessary damage from continued exposure to the cryoprotectant. Motility, vitality and DNA integrity were then measured. For certain studies, sperm separation involved the Felix™ System (Memphasys, Sydney, Australia), as described below.

### Cryopreservative evaluation

Since both ethylene glycol and glycerol are effective cryoprotectants [20, 21] but have different physico-chemical properties, we combined these reagents in our cryoprotectant mixture in a ratio of 1:1. In addition, we added a small amount of dimethyl sulfoxide (DMSO) given its proven track record as a cryoprotectant and its capacity to impede free radical formation [22, 23]. The consequences of varying the amount of cryoprotectant in the medium were then assessed as follows: 1.5 mL of semen samples were equally aliquoted (250 µL semen mixed with 250 µL medium) into 6 Eppendorf’s. The media tested included: Quinn’s (control); 100% carrier medium (0% cryoprotectant); 98.25% test medium, 0.56% glycerol, 0.56% ethylene glycol, and 0.13% dimethyl sulfoxide (DMSO) (1.25% cryoprotectant); 97.5% test medium, 1.13% glycerol, 1.13% ethylene glycol, and 0.25% DMSO (2.5% cryoprotectant); 95% test medium, 2.25% glycerol, 2.25% ethylene glycol, and 0.5% DMSO (5% cryoprotectant); and 90% test medium, 4.5% glycerol, 4.5% ethylene glycol, and 1% DMSO (l0% cryoprotectant). All Eppendorf’s were then placed into straws and frozen as described above.

### Production of novel cryopreservation carrier medium (CCM)

The carrier media employed in this study were either a balanced salt solution that is known to support human sperm function (medium BWW) or a novel cryopreservation carrier medium (CCM) designed with the primary intention of avoiding NaCl as the major osmolyte [24, 25] as presented in Table 1. For these CCM development studies, highly purified sperm populations were generated following Percoll-gradient centrifugation as described [26].

**Table 1:** Composition of the cryopreservation base medium (CBM) used in these studies

Constituent	Amount
CBM composition (50 mL)	
Polyvinyl alcohol	100 mg (2 mg/mL)
Sodium hydrogen carbonate	84.007 mg (20 mM)
D-glucose	150 mg (3 mg/mL)
Histidine	1.123 g
Taurine	302 mg
Pentoxifylline	69.578 mg (5 mM)
Gentamicin	500 µL (0.5 mg/mL)
Penicillin–streptomycin	500 µL
1 M HEPES	1 mL (20 mM)
1 M MOPS	1 mL (20 mM)
Make up to volume 50 mL using NaCl-free stock	
NaCl-free stock composition (1L)	
Potassium chloride (KCl)	356 mg
Calcium chloride dihydrate (CaCl_2_.2H_2_O)	250 mg
Potassium phosphate monobasic (KH_2_PO_4_)	162 mg
Magnesium sulfate heptahydrate (MgSO_4_.7H_2_O)	294 mg
Make up to 1L with Milli Q water	

### Variations to CCM

A variety of changes were made to CCM in order to improve its performance (*n* = 5–10). For these experiments, semen samples were equally aliquoted (250 µL semen mixed with 250 µL medium) into Eppendorf tubes. The changes investigated included the addition of vitamin C (0.1, 0.2, 0.4 or 0.8 mM; L-ascorbic acid, Sigma-Aldrich, Burlington, MA, USA), the addition of ethylenediaminetetraacetic acid (EDTA) (0.5, 1 or 2 mM; Sigma-Aldrich, Burlington, MA, USA), reduction of calcium chloride (1.7 mM, 0.85 mM, 0.42 mM; Sigma-Aldrich, Burlington, MA, USA); addition of crocin (0.5, 1 or 2 mM; Sigma-Aldrich, Burlington, MA, USA); addition of zinc sulfate (25, 50 or 100 µM; Sigma-Aldrich, Burlington, MA, USA); addition of L-ergothioneine (4, 6 or 8 mM: Sigma-Aldrich, Burlington, MA, USA) or the addition of *myo*-inositol (5.5, 11, 22, 44 or 66 mM; Sigma-Aldrich, Burlington, MA, USA). In addition, we investigated the replacement of DMSO with Cyrene™ (Sigma-Aldrich, Burlington, MA, USA).

### Post thaw assessments under clinical conditions

A total of 0.5 mL aliquots of semen (*n* = 18) were equally aliquoted into 2 Eppendorf tubes: 250 µL mixed with either 250 µL Quinn’s Medium (control) or 250 mL mixed with 250 µL modified CCM. All Eppendorf’s were then placed into straws and frozen. Upon thawing, samples were diluted 1:1 with BWW, then isolated from the semen mixture via electrophoretic separation with the Felix™ System (Memphasys Ltd, Sydney, Australia) as described [27]. Once isolated and resuspended in BWW, the spermatozoa were assessed for motility, vitality, and DNA damage.

### Analytical procedures

**Fig. 1:**
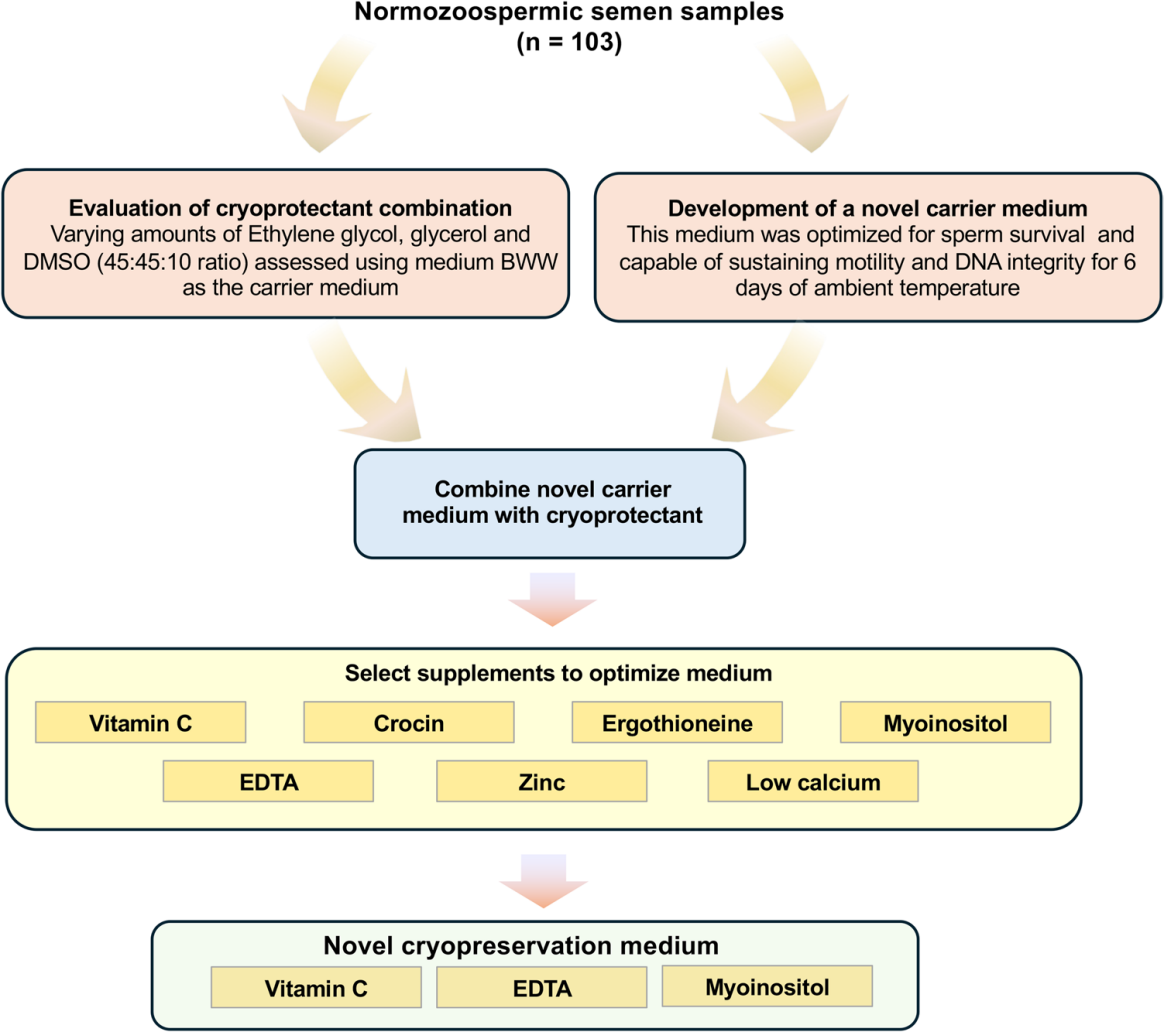
A flowchart illustrating the project design

Osmolarity was measured by freezing point depression using a single sample micro-osmometer (Advanced™ Instruments, Single-Sample Micro-Osmometer, Advanced Model Osmo1; John Morris Group, Sydney, Australia). A sperm chromatin-dispersion assay (HALO) was performed on frozen-thawed samples to determine the levels of DNA fragmentation in the spermatozoa. Samples were spread on precoated agarose slides and covered for various lengths of time in: HCl, Tris buffer 1 containing DTT, Tris buffer 2, Tris-Boric Acid-EDTA buffer, increasing strengths of ethanol, 4′,6-diamidine-2′-phenylindole dihydrochloride (DAPI, Sigma-Aldrich, Burlington, MA, USA) and PBS; Mowoil® and cover slips were then applied. The exact constitutions of each solution and the incubation times used, are as described [6]. Spermatozoa were assessed for a ‘halo’ of fluorescent DNA under fluorescent microscopy at × 400 magnification. The presence of a ‘halo’ indicated undamaged DNA, while its absence indicated pre-existing single-strand breaks. For scoring purposes, cells classified with large or medium halos were considered healthy, while cells with small to no halo, or were too degraded to fluoresce properly, were considered damaged. The overall study design is presented in Fig. 1.

### Statistical analysis

Statical analysis of the data was undertaken using the JMP Pro 17 statistical software package (SAS Institute, Cary, NC, USA). All data are presented as the mean ± the standard error. Datasets were analyzed by one- and two-way ANOVA, paired *t*-test, and linear regression following an initial screen with the Anderson–Darling test to determine the normality of data distribution. Data that did not conform to a normal distribution were transformed using the Box-Cox transformation with optimized lambda values calculated for each parameter. In the figures, all transformed data are presented in their original form for easier interpretation. Throughout, the minimum level of statistical significance was set at *P* < 0.05.

## Results

### Initial assessment of cryoprotectant combination

**Fig. 2:**
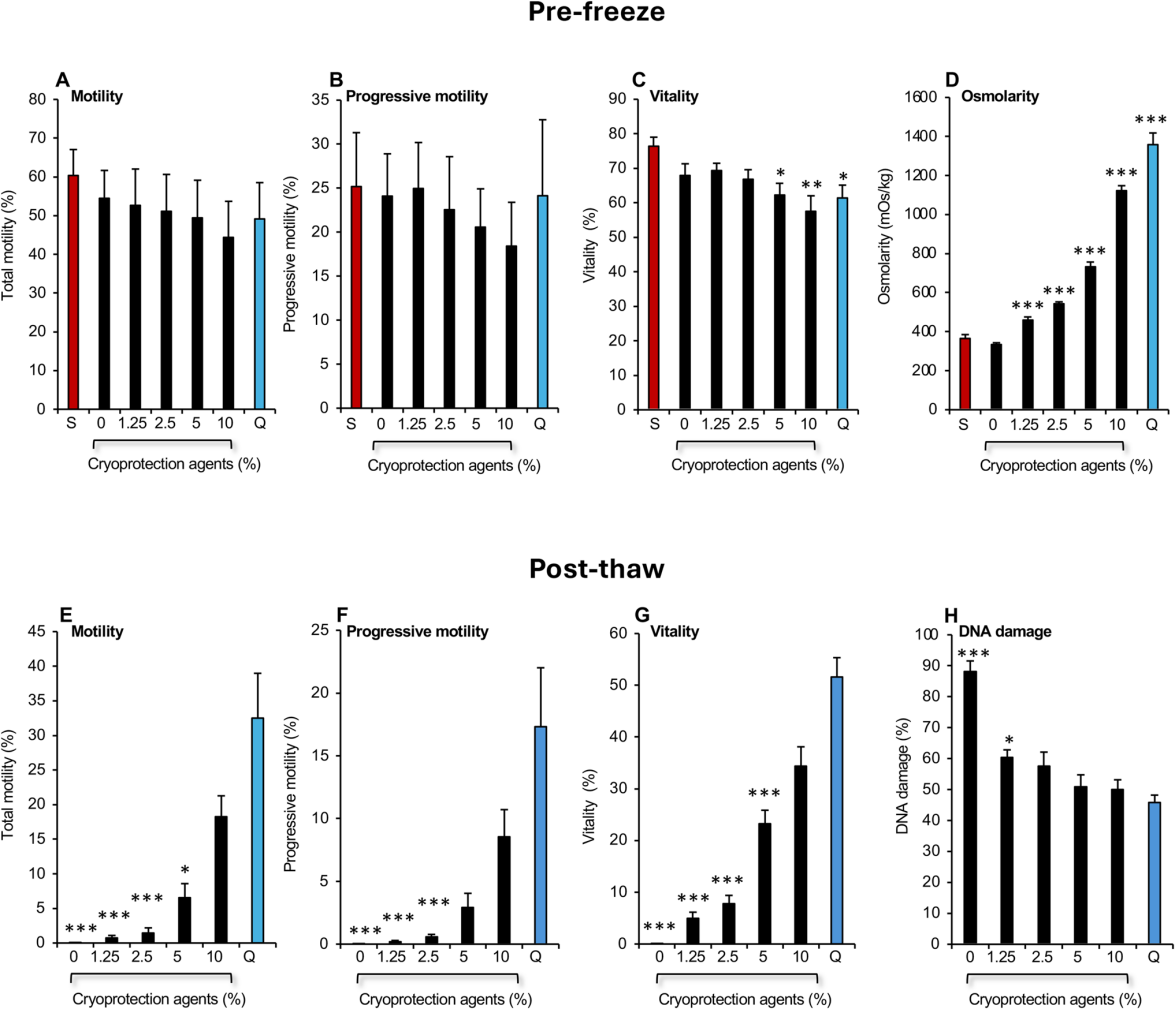
Impact of cryopreservation agents on human spermatozoa. **A**–**D** Addition of a cryoprotectant mixture (ethylene glycol, glycerol and DMSO in ratios of 45:45:10) 1:1 with human semen in doses ranging from 1.25 to 10% (v/v) at room temperature using medium BWW as the carrier (*n* = 5; *P < 0.05; **P < 001; ***P < 0.001 relative to the original semen sample, S). **A** Total motility; **B** progressive motility; **C** vitality; **D** osmolarity. **E**–**H** The post thaw qualities of the spermatozoa when the above samples were subjected to cryopreservation. (*n* = 5; *P < 0.05; **P < 001; ***P < 0.001 relative to the performance of Quinn’s Advantage™ Sperm Freezing Medium, Q) All data presented as mean ± SEM

This study was initiated with an assessment of the performance of our cryoprotectant combination (ethylene glycol, glycerol and DMSO in a 45:45:10 ratio) in doses ranging from 1.25 to 10% (v/v), using a balanced salt solution (BWW) supplemented with 1 mg/mL polyvinyl alcohol as the base medium and Quinn’s Advantage™ Sperm Freezing Medium as the positive control. The addition of cryoprotectant to semen at room temperature had no significant impact on either motility or progressive motility, although the vitality of the spermatozoa was slightly compromised in Quinn’s and when the test cryoprotectant combination exceeded 5% (Fig. 2A–C). This loss of vitality was observed when the osmolarity of the cryopreservation mixture exceeded 700 mOs/kg, as observed with 5% and 10% cryoprotectant as well as Quinn’s medium (Fig. 2C, D). Moreover, across the entire dataset, sperm vitality and osmolarity exhibited a highly significant negative linear correlation *(P* < 0.001; *R*^2^ = 0.31; *n* = 35).

Despite the negative impact of hyperosmotic cryoprotectant media on sperm viability, these agents were capable of some level of protection of the spermatozoa from cryoinjury. Thus, following cryopreservation of the above samples, the retention of total motility, progressive motility, and vitality was positively correlated with the amount of cryoprotectant added such at 10% these parameters of sperm quality were not significantly different from Quinn’s cryopreservation medium, although they were still clearly inferior to the latter (Fig. 2E–G). In the same dataset, there was a negative association between sperm DNA damage and the amount of cryoprotectant added such that the loss of genetic integrity was not significantly different from Quinn’s Advantage™ medium when the amount of cryoprotectant added to the medium exceeded 2.5% (Fig. 2H), although the mean levels of DNA damage were still slightly higher. These data demonstrated the effectiveness of our cryoprotectant combination; however, the use of a balanced salt solution as the carrier medium for these reagents was clearly damaging to the cells. An alternative approach was needed that avoided the use of NaCl as a major osmolyte.

### Creation of a novel cryopreservation carrier medium

**Fig. 3:**
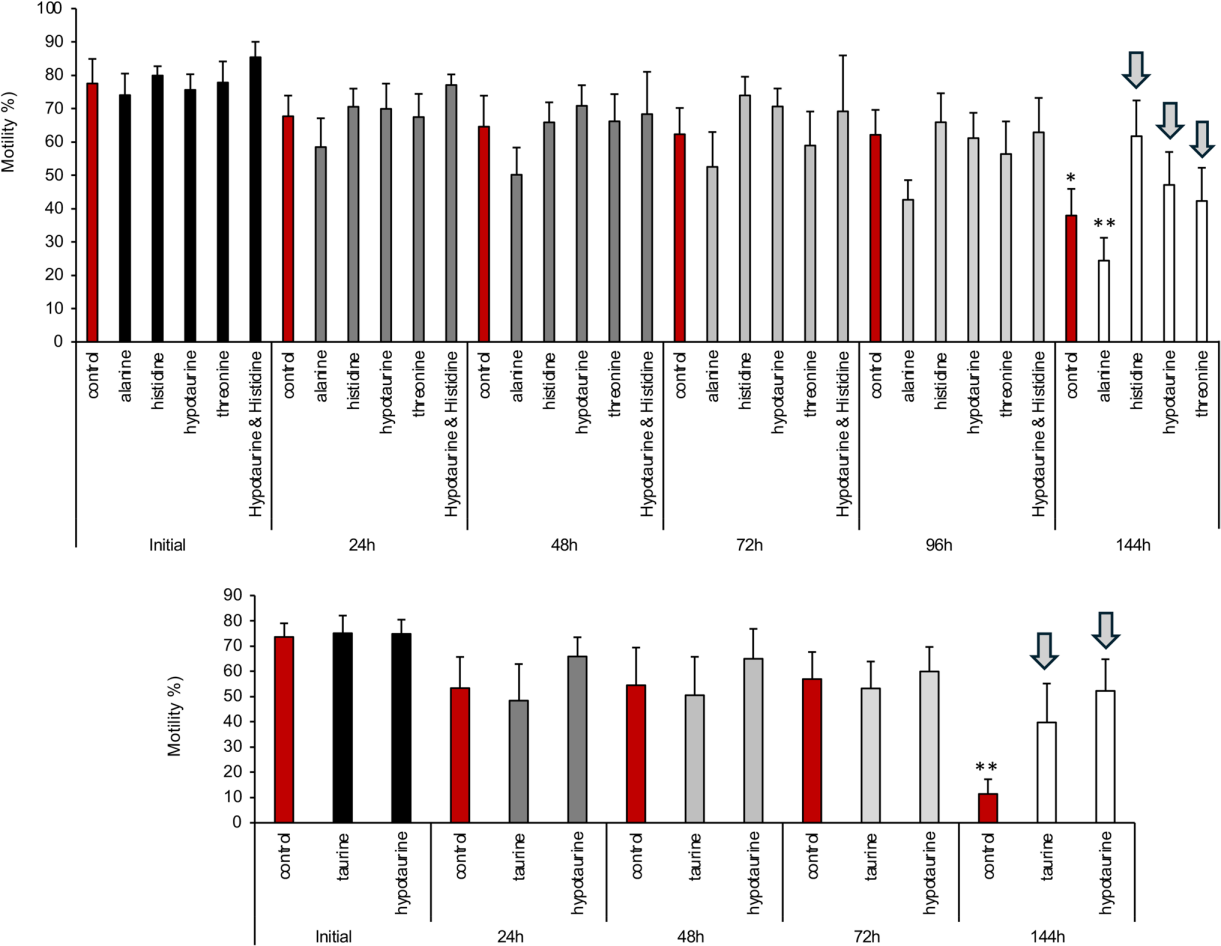
Examples of the amino acid screen used to determine the optimal amino acids to be used to supplement medium CCM in place of NaCl. **A** All amino acids were suspended in NaCl-free CMM at a dose, 1.65 mM, needed to sustain medium osmolarity at 310 mOs/kg. Sperm motility was followed over a 144-h period at ambient temperature. Several amino acids exhibited an ability to support sperm movement, consistent examples being histidine, hypotaurine, taurine, and threonine (arrowed); by contrast motility significantly declined in the unsupplemented controls and in the presence of alanine. **B** repeat analysis focusing on a comparison of taurine and hypotaurine, again demonstrating that these amino acids could preserve sperm motility over 6 days in contrast to the unsupplemented controls All data presented as mean ± SEM,* n* = 4–6 in each group (*P < 0.05; **P < 001). The combination of histidine and taurine was ultimately selected to form the basis of CCM

Our starting point for the development of a novel cryopreservation carrier medium (CCM) began with the balanced salt mixture characteristic of medium BWW (KCl, CaCl_2_**.**2H_2_O, KH_2_PO_4_, MgSO_4_**.**7H_2_O and NaHCO_*3*_) but with NaCl omitted. This medium is known to be highly supportive of sperm function particularly when supplemented with a macromolecule such as PVA [28] and a glycolysable substrate in the form of glucose (Table 1). The carrier medium was also double-buffered with HEPES (20 mM) and MOPS (20 mM) to maintain pH in the optimal range and contained a phosphodiesterase inhibitor (pentoxifylline) to raise intracellular levels of cAMP [28]. In order to replace NaCl with an osmolyte that the cells would not have to expend energy to control, we examined 16 amino acids (proline, threonine, alanine, glycine, glutamine, histidine, glycine, isoleucine, phenylalanine, serine, valine, taurine, hypotaurine, tyrosine, lysine, arginine) for their ability to serve as an osmolyte for CCM. These amino acids were added to the NaCl-free medium BWW and the resulting media used to incubate Percoll-purified spermatozoa over a period of 6 days at ambient temperature. The ability of the media to support human sperm function was determined on the basis of sperm motility. The amino acids were employed at a dose (1.65 mM) needed to restore the osmolarity of the NaCl-free to 310 mOs/kg. Examples of these amino acid screens are given in Fig. 3A. Two-way analysis of variance revealed a highly significant difference due to both treatment (*P* < 0.001) and time (*P* < 0.001) but no significant interaction between these criteria. Of all the amino acids tested, histidine, threonine and taurine or hypotaurine were found to consistently support levels of motility that did not change significantly over 6 days of in vitro culture, while motility significantly declined in both control incubations with no amino acid supplementation (*P* < 0.05) and in the presence of alanine (*P* < 0.01; Fig. 3A). A repeat analysis focusing on a taurine and hypotaurine again revealed that while sperm motility declined significantly over 6 days (*P* < 0.01), in the presence of these amino acids, no significant decline in motility was observed (Fig. 3B).

### CCM the basis for a cryopreservation medium

**Fig. 4:**
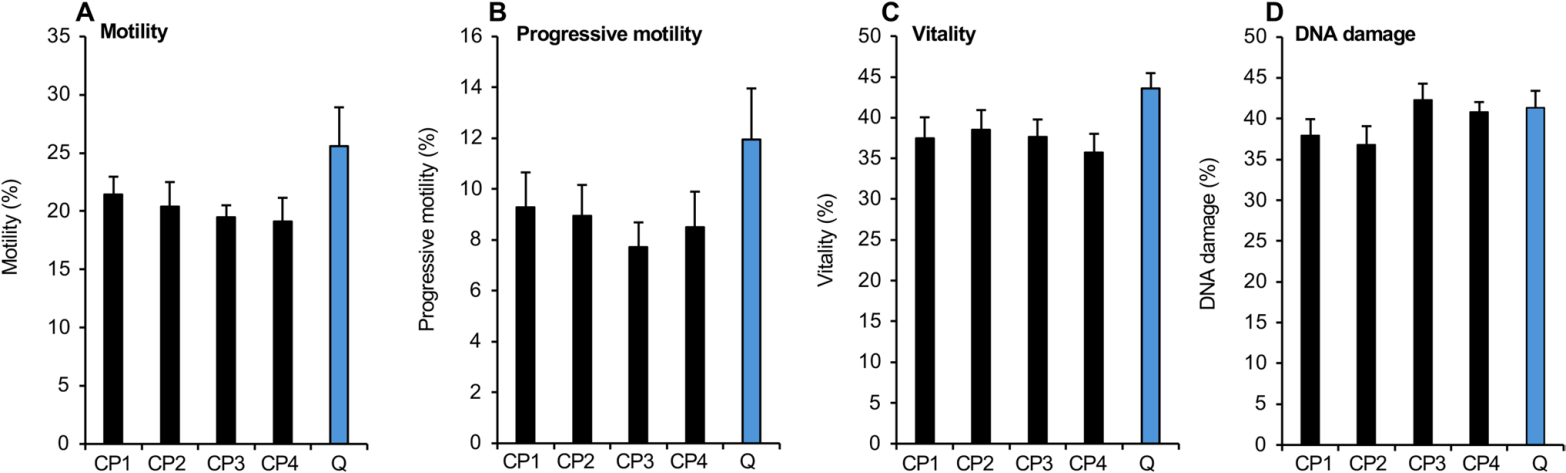
Impact of cryoprotectant supplementation of CCM on post thaw properties of spermatozoa relative to Quinn’s Advantage™ Sperm Freezing Medium, Q. Medium CCM was supplemented with 10% (v/v) of a cryoprotectant blend containing ethylene glycol, glycerol and DMSO in the following combinations: CP1 4.5%:4.5%:1%; CP2 6%:3%:1%; CP3 7%:2%:1%; CP4 8.5%:1%:0.5% (*n* = 12). All data presented as mean ± SEM

Given the exceptional ability of CCM to support sperm function, we next sought to determine how this medium would perform as a cryopreservation medium. For this purpose, we assessed post-thaw semen quality in a group of 12 samples that had been cryostored in CCM supplemented with 10% cryoprotectant. The latter comprised different combinations of ethylene glycol, glycerol, and DMSO in the following relative amounts: (CP1) 4.5%:4.5%:1%, (CP2) 6%:3%:1%, (CP3) 7%:2%:1%, and (CP4) 8.5%:1%:0.5%. Following cryopreservation, no significant differences were observed in the levels of vitality, motility, and DNA integrity exhibited by semen samples cryopreserved in commercial Quinn’s Advantage™ medium or any of the combinations of cryoprotectant added to CCM (Fig. 4). Further modifications of the medium were clearly required if we were to improve significantly on a commercial product such as Quinn’s. As there were no significant differences between any of the cryoprotectant combinations used, we opted to use CCM supplemented with 10% cryopreservation agent in the form of ethylene glycol: glycerol: DMSO in the ratio 4.5%:4.5%:1% as a basis for the further development of this medium.

### Modification of CCM to enhance its performance as a cryopreservative

**Fig. 5:**
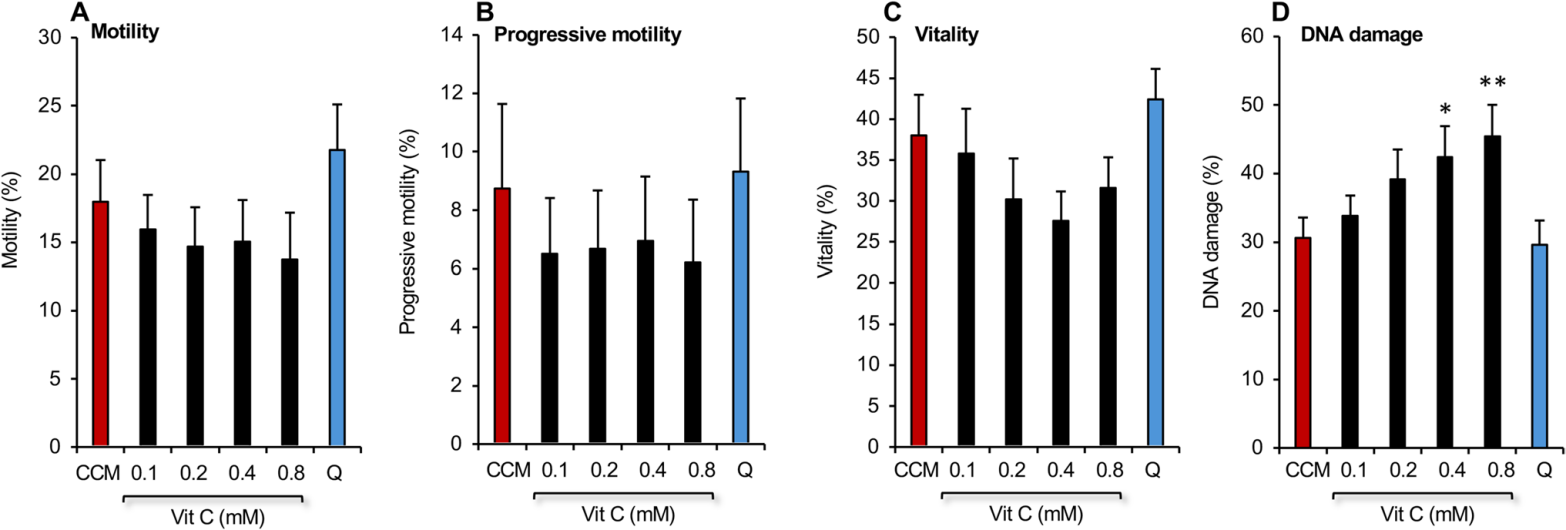
Impact of adding different doses of vitamin C to CCM on the post thaw properties of human spermatozoa relative to Quinn’s Advantage™ Sperm Freezing Medium. (*n* = 5; *P < 0.05; **P < 001 relative to the performance of Quinn’s, Q). All data presented as mean ± SEM

**Fig. 6:**
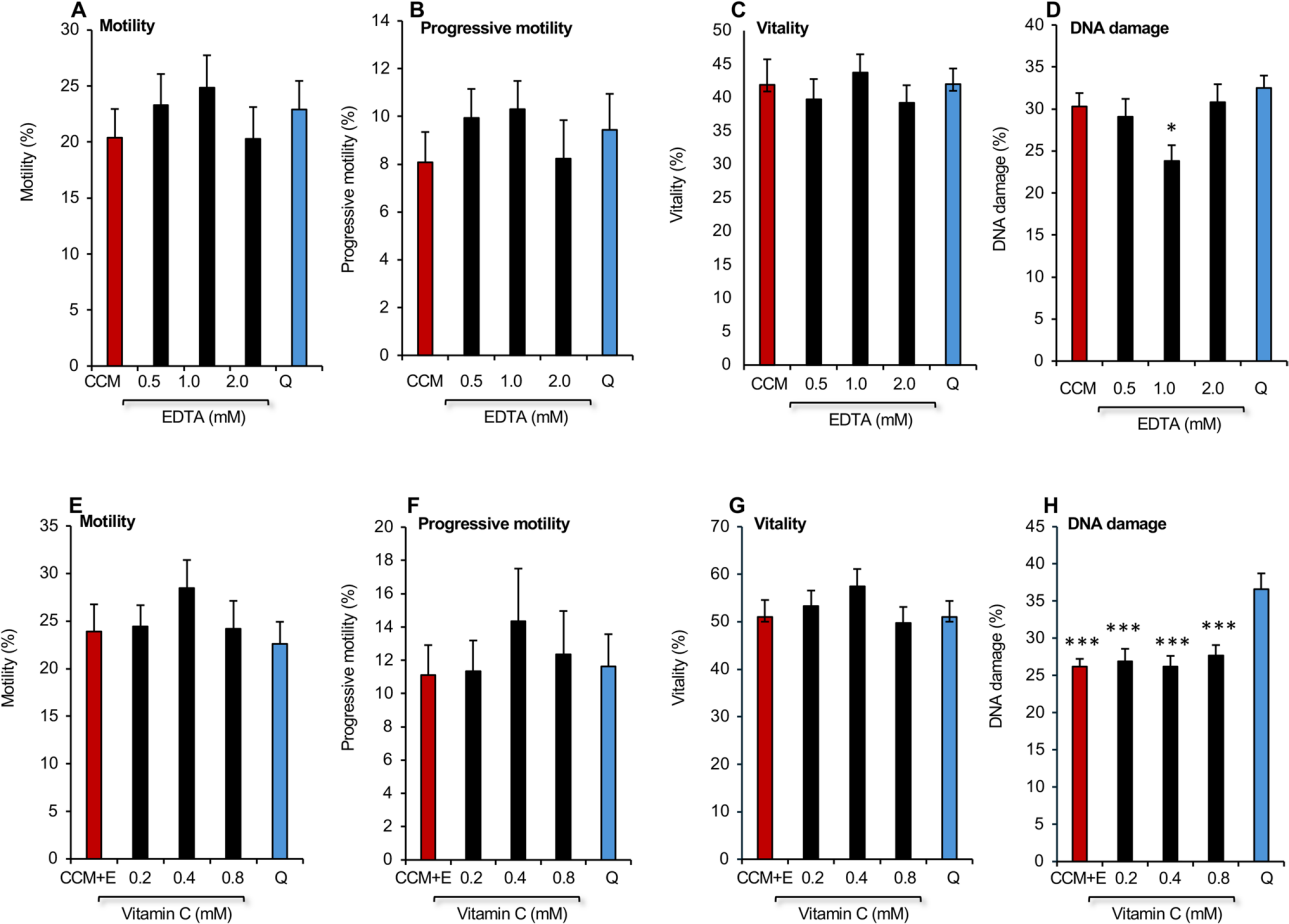
Impact of adding EDTA alone and in combination with vitamin C to CCM on the post thaw properties of spermatozoa relative to Quinn’s Advantage™ Sperm Freezing Medium, Q. **A**–**D** Addition of EDTA alone. (*n* = 5; *P < 0.05 relative to the performance of Quinn’s, Q). **E**–**H** Addition of vitamin C to CCM supplemented with 1 mM EDTA, CCM + E (*n* = 10; ***P < 0.001 relative to Quinn’s, Q). All data presented as mean ± SEM

Since we have recently demonstrated the ability of vitamin C to enhance the performance of commercial cryopreservation media [12], we examined the consequences of adding vitamin C (0.1–0.8 mM) to CCM. Following cryostorage, the addition of vitamin C was found to induce a surprising non-significant downward trend in motility, progressive motility, and vitality. This trend was in turn associated with a significant increase in DNA damage (*P* < 0.05 for the overall effect of treatment by ANOVA) which was particularly marked at the highest doses of 0.4 (*P* < 0.05) and 0.8 mM (*P* < 0.01) (Fig. 5). Since reducing agents such as vitamin C can exert pro-oxidant effects on cells by reducing trace amounts of transition metals such as iron and copper, thereby accelerating redox cycling activity, ROS generation, and oxidative damage [26], we next examined the impact of EDTA, an effective chelator of such metals. A dose-dependent analysis of EDTA (0.5–2.0 mM) revealed no significant impact on motility, progressive motility, and vitality; however, the 1 mM dose induced a significant decline in DNA damage (*P* < 0.05 for the overall effect of treatment by ANOVA) (Fig. 6A–D).

In the next analysis we combined different doses of vitamin C with the EDTA (1 mM). This analysis confirmed the ability of EDTA addition to effectively suppress DNA damage, although the addition of vitamin C did not lead to any further improvement in DNA integrity (Fig. 6E–H). However, the addition of vitamin C to EDTA supplemented medium did improve motility, progressive motility and vitality, particularly at the 0.4 mM dose, although the degree of increase did not reach statistical significance in this limited data set. Nevertheless, when we expanded the group to *n* = 28 and focused on a comparison between Quinn’s and CCM supplemented with EDTA + 0.4 mM vitamin C (CCM + EC), the latter was associated with significant increases in both motility (22.1 ± 1.3% in Quinn’s vs 27.3 ± 1.6% in CCM + EC; *P* < 0.05) and vitality (48 ± 1.7% in Quinn’s vs 53.7 ± 1.7% in CCM + EC; *P* < 0.05) as well as a significant decline in DNA damage (34.1 ± 1.7% in Quinn’s vs 24.7 ± 1.6% in CCM + EC; *P* < 0.001).

**Fig. 7:**
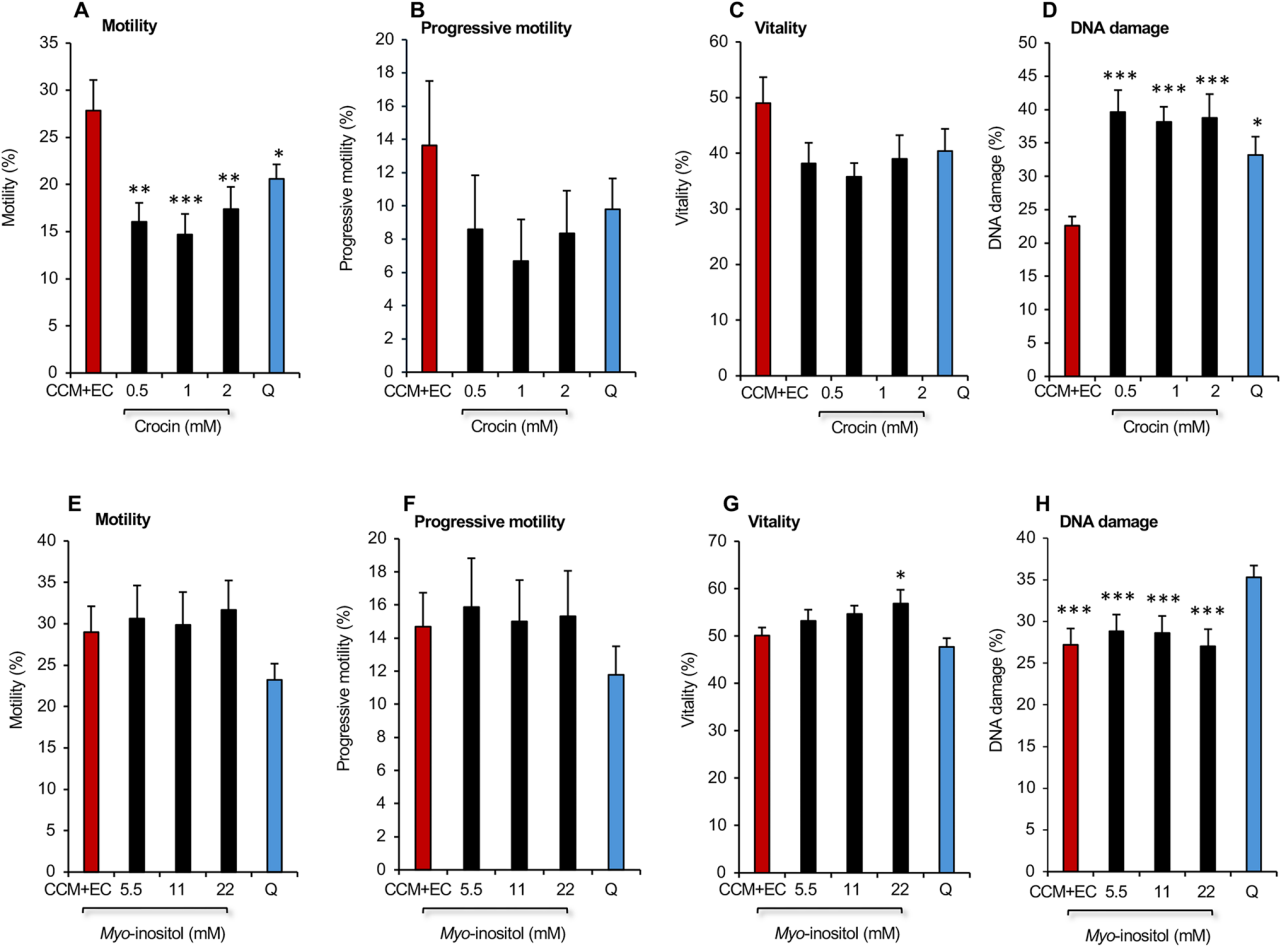
Impact of adding the supplements crocin and myoinositol to CCM incorporating EDTA and vitamin C (CCM + EC) on the post thaw properties of spermatozoa in comparison with Quinn’s Advantage™ Sperm Freezing Medium, Q. **A**–**D** Consequences of adding crocin to the cryopreservation medium (*n* = 5; *P < 0.05; **P < 0.01; ***P < 0.001 relative to the control, CCM + EC). **E**–**H** Impact of adding *myo*-inositol to the cryopreservation medium. (*n* = 5; *P < 0.05; ***P < 0.001 relative to Quinn’s). All data presented as mean ± SEM

In light of the observation that sperm motility is suppressed by high levels of calcium in the ejaculate [29] we next sought to further refine this medium by reducing its calcium content. This analysis demonstrated that reducing the calcium content of the CCM + EC medium did not improve its effectiveness as a cryopreservation medium (Supplementary Fig. 1). Indeed, halving the calcium content from 1.7 mM to 0.8 mM significantly increased levels of DNA damage (*P* < 0.01) so that it was not significantly different from Quinn’s (Supplementary Fig. 1). Similarly, following a recent report indicating that the antioxidant properties of zinc could improve the cryopreservation of human spermatozoa [30], we examined the ability of this cation to increase the effectiveness of CCM + EC as a freezing medium, but observed no significant impact on sperm movement, vitality, or DNA integrity. (Supplementary Fig. 2). Addition of the powerful antioxidant, crocin, which has also recently been shown to support the cryopreservation of human spermatozoa [16], actually increased levels of DNA damage seen in frozen-thawed cells (*P* < 0.001; Fig. 7A–D). In addition, this reagent significantly suppressed sperm motility (*P* < 0.01 for the overall effect of treatment by ANOVA; Fig. 7A), while having no beneficial effect on sperm vitality (Fig. 7C). Addition of yet another antioxidant, ergothioneine, (a thiolated derivative of histidine with antioxidant properties which has been shown to protect the spermatozoa of several species from cryoinjury [31]) to CCM + EC, also had no significant impact on the effectiveness of the cryopreservation process as applied to human spermatozoa (Supplementary Fig. 3).

The only reagent that appeared to increase the effectiveness of CCM + EC was *myo*-inositol. In a dose-dependent study, we showed that while this compound did not improve the motility or progressive motility of frozen-thawed human spermatozoa, it did significantly improve the vitality of these cells (*P* < 0.05 for the overall effect of treatment by ANOVA) relative to both Quinn’s medium (*P* < 0.01) and CCM + EC (*P* < 0.05) (Fig. 7E–H) at the 22 mM dose. DNA integrity was significantly lower with CCM + EC compared with Quinn’s Advantage™ medium, but this protective action was not improved by *myo*-inositol. The value of this compound lay entirely with its positive impact on vitality (Fig. 7E–H). Increasing the dose of *myo*-inositol to as high as 66 mM did not further improve its ability to support sperm viability or any other parameter of sperm quality (data not shown). The final evaluation undertaken to optimize this cryopreservation medium was to determine whether Cyrene™ (dihydrolevoglucosenone) an aprotic, dipolar, small molecular mass solvent that has been recommended as a less toxic substitute for DMSO [32], could substitute for the latter as a cryoprotection agent. Changing 1% DMSO to 1% Cyrene™ did not improve the performance of the protection medium in terms of sperm motility and vitality and elevated DNA damage to the point that it was similar to Quinn’s (Supplementary Fig. 4). Considering these data, it was decided to retain DMSO as a component of the cryopreservation medium and not to replace it with Cyrene™.

### Cryopreservation and sperm isolation

The optimized cryoprotection medium generated in this study therefore comprised the NaCl-free carrier medium (CCM) supplemented with 10% cryoprotection medium (ethylene glycol, 4.5%: glycerol, 4.5%: DMSO, 1%), EDTA (1 mM), vitamin C (0.4 mM) and *myo*-inositol (22 mM). In the following section this formulation is referred to as ‘CCM complete’. Evaluation of this medium as a cryoprotectant was undertaken on 20 independent samples that were frozen with either CCM complete medium or Quinn’s Advantage™ sperm freezing medium and then thawed. This analysis revealed that sperm populations frozen and then thawed in CCM complete were consistently superior to Quinn’s with respect to total motility (27.2 ± 2.3% vs 21.4 ± 1.5%; *P* < 0.05), vitality (54.1 ± 2.0% vs 47.7 ± 1.6%; *P* < 0.05), and DNA damage (25.7 ± 1.6% vs 33.5 ± 1.2%; *P* < 0.001); only in the case of progressive motility did the difference not reach statistical significance (12.5 ± 1.7 vs 11.1 ± 1.2%).

**Fig. 8:**
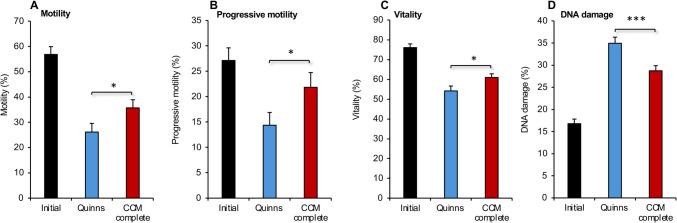
Comparison of the complete CCM medium with Quinn’s Advantage™ Sperm Freezing Medium following cryopreservation and subsequent isolation of spermatozoa with the Felix™ System. All data presented as mean ± SEM; (*n* = 18; *P < 0.05; ***P < 0.001) for the difference between Quinn’s medium and CCM complete

To see how these cryopreservation media might compare when spermatozoa were subsequently isolated for assisted conception procedures, 18 independent samples were cryopreserved, thawed, and then processed by the Felix™ System [27]. The results of this analysis are presented in Fig. 8 in comparison with the quality of the original semen sample. As anticipated, the motility, progressive motility, and vitality of spermatozoa isolated using the Felix™ System, was significantly impacted by the cryostorage process (*P* < 0.001 for the overall effect of treatment by ANOVA; Fig. 8A–C). However, all these parameters were significantly greater in CCM complete compared with Quinn’s Advantage™ medium (*P* < 0.05; Fig. 8A–C). Similarly, cryopreservation dramatically impacted DNA integrity (*P* < 0.001 for the overall effect of treatment by ANOVA; Fig. 8D) and in this case the difference between CCM complete and Quinn’s Advantage™ medium was highly significant (*P* < 0.001; Fig. 8D).

## Discussion

This study has not only defined a sperm preservation medium that can sustain purified populations of human spermatozoa at ambient temperature for up to 6 days, but also served as the basis for a cryopreservation medium that represents a statistically significant improvement over current formulations in clinical use, in terms of the maintenance of sperm movement, vitality, and DNA integrity (Fig. 8).

The basic carrier medium (CCM) was founded on BWW, a medium that has been repeatedly validated for its capacity to support human sperm function since its introduction in 1971 [19, 33]. However, for long-term storage purposes we removed the NaCl from this medium so that spermatozoa would not have to expend energy actively driving their Na^+^/K^+^ ATPases in order to maintain an appropriate intracellular ionic balance. Of course, some Na^+^ is required for normal sperm function in order to regulate membrane potential and drive Na +/H + exchange activity [34, 35]; however, Na^+^ overloading is associated with ATP depletion and a loss of sperm motility [35]. The small amount of sodium needed to sustain cellular function was therefore provided predominantly by the NaHCO_3_ component of the medium, while the need to maintain osmotic balance was met by the incorporation of amino acids. We assessed several amino acids for this role and selected histidine for its capacity to consistently support sperm motility over prolonged periods of time (Fig. 3). Histidine may well be important for the maintenance of human sperm function because of its ability to counteract ROS generation and effect the adduction of cytotoxic aldehydes, such as 4-hydroxynonenal, generated by spermatozoa as a consequence of lipid peroxidation and the promotion of cell senescence [36, 37]. For the purpose of generating an effective sperm storage medium, the histidine was supplemented with taurine, given the abundance of evidence that this amino acid is supportive of sperm function in a wide variety of species and can aid the survivability of these cells in vitro [38–41]. In addition to amino acid supplementation, the storage medium was double buffered with MOPS and HEPES to maintain an optimal pH of around 7.6–7.8 [42] and contained pentoxifylline to enhance the cAMP content of the stored cells and thereby sustain their motility and fertilizing potential [33]. We wished to generate a defined medium lacking any expensive, undefined constituents such as albumin, so we elected to provide macromolecular support to the spermatozoa in the form of PVA [44]. In combination, these reagents provided us with a basic medium that could support human sperm motility for up to 6 days in vitro at ambient temperature (Fig. 3). To determine whether this ambient temperature medium could also serve as the basis for a novel sperm freezing medium, we needed to explore the impact of adding cryoprotective agents.

In order to undertake these studies, we examined a combination of cryoprotective agents (glycerol, ethylene glycol and DMSO) each one of which has been shown to be effective in its own right. DMSO is an extremely effective inhibitor of free radical formation and the prevention of intracellular ice formation; glycerol is a well-established membrane permeant cryoprotection agent capable of reducing the intracellular freezing point, while ethylene glycol has a lower molecular mass than glycerol and penetrates cells more rapidly. Since the chemical properties, and toxicity, of each of these cryoprotective agents are different, we followed the lead of other researchers in using them in combination [45]. The addition of cryoprotectant was found to be essential for the cryopreservation of human spermatozoa (Fig. 2) and the impact was clearly dose dependent with optimal levels of support being observed when the cryoprotective supplement reached 10% (v/v). Substituting Cyrene™ for DMSO in an effort to reduce the toxicity did not materially affect the outcome of the cryopreservation process (Supplementary Fig. 4). The carrier for this combination of cryoprotective agents was however found to be critical and clearly conventional medium BWW did not fulfil this role (Fig. 2). However, when modified to create the basal carrier medium, CCM, the addition of cryoprotectants resulted in levels of post-thaw motility and DNA damage that were no different from Quinn’s Advantage™ medium, regardless of the ratios of glycerol, ethylene glycol, and DMSO used in the cryoprotective mixture (Fig. 4).

Settling on a cryoprotectant formulation containing 4.5% glycerol, 4.5% ethylene glycol and 1% DMSO, we next sought to improve the performance of CCM by making a number of changes to its composition. Addition of vitamin C did not enhance motility but at the highest doses tested actually increased DNA damage, possibly due to the reduction of contaminating transitional metals, such as iron [26] (Fig. 5). The addition of EDTA alone had little impact on sperm motility but did significantly reduce DNA damage at a 1 mM dose in keeping with previous results in other species [46–48]. Addition of vitamin C did not further enhance the ability of EDTA to suppress DNA fragmentation (Fig. 6); however, it did enhance sperm motility to a level that represented a statistical improvement over Quinn’s Advantage™ medium in an expanded group of 28 samples. Lowering the calcium content of the medium was also assessed [49–51] but proved unhelpful as it reduced sperm motility and significantly increased levels of sperm DNA damage (Supplementary Fig. 1). Similarly, attempts to increase the antioxidant capacity of CCM using crocin, ergothioneine, or zinc all failed to deliver significant improvements to the cryopreservation potential of CCM + EC (Fig. 7; Supplementary Figs. 2, 3) despite literature suggesting that all of these additives should have a beneficial impact on the freezing of human spermatozoa [16, 30, 31]. It is possible that the presence of vitamin C together with the powerful antioxidants that are already present in human seminal plasma [52] provide adequate protection to the spermatozoa against oxidative stress. The addition of yet more antioxidant support in the form of crocin, ergothioneine and zinc just leads to a state of redox imbalance characterized by reductive stress [53], which can be just as damaging as its oxidative counterpart.

The next supplement to be assessed for its capacity to improve CCM + EC was *myo*-inositol. *Myo*-inositol is a member of the vitamin B complex group, involved in multiple aspects of cell biology from membrane signalling to protein expression. In spermatozoa specifically, it is involved in motility, maturation, capacitation, and acrosome reaction and when added as a supplement, has been shown to improve these functions as well as vitality and DNA integrity scores [54–56]. In this study, *myo*-inositol did not improve sperm motility or the low levels of DNA damage already observed with CCM + EC; however, it did improve sperm vitality (Fig. 7). A possible mechanism for this effect might be suppression of the truncated intrinsic apoptotic pathway responsible for the mediation of sperm senescence [57]. Spermatozoa are prevented from undergoing apoptosis by the PI3 (phosphoinositide 3) kinase/AKT pathway which induces the phosphorylation and inactivation of Bad, a pro-apoptotic Bcl-XL regulator [57]. Since *myo*-inositol is the structural base for inositol-triphosphate, it may be enhancing vitality by reducing apoptosis.

The limitations of this study include the group size, which was adequate to demonstrate statistical significance and reflects the reality of semen sample availability, the low levels of variation between individuals in our donor cohort and the number of biological replicates typically employed in studies of human spermatozoa. Further validation of the sperm storage medium defined in this study will ultimately be required using larger cohorts of subjects, including the kind of sub-fertile males encountered in a clinical environment, before clinical implementation can be recommended. In addition, the Halo assay used to determine DNA damage has inherent limitations because it is an indirect assay that does not discriminate between single and double DNA strand breaks, does not reflect other forms of DNA damage, possesses a relatively subjective endpoint, and is sensitive to variations in sample handling.

## Conclusions

When spermatozoa were cryopreserved using the CCM complete medium, incorporating vitamin C, EDTA and myoinositol, and then prepared for assisted conception using a commercial sperm preparation device, the Felix™ System [27], sperm populations were generated that were significantly more motile and vital than those recovered following cryopreservation in Quinn’s Advantage™ medium, while the levels of sperm DNA damage were considerably reduced. The ability of our novel cryopreservation medium to retain such high levels of motility, vitality and DNA integrity represents a significant improvement over the *status quo*, particularly for men requiring cryopreservation due to illness or subfertility, where the spermatozoa are extremely vulnerable to cryoinjury [58, 59]. This novel preservation medium, has great potential in terms of cost, safety and efficacy for both ambient temperature storage and cryopreservation. While the scientific rationale behind this medium has been statistically demonstrated using donor samples, further studies will now be required on large patient cohorts, as a prelude to clinical implementation.

## Supplementary Information

Below is the link to the electronic supplementary material.

Supplementary file1 (PPTX 109 KB)

## Data Availability

The data presented in this study are available in the article.

## References

[1] Aitken RJ. What is driving the global decline of human fertility? Need for a multidisciplinary approach to the underlying mechanisms. Front Reprod Health. 2024;6:1364352. 10.3389/frph.2024.1364352. 38726051 10.3389/frph.2024.1364352 PMC11079147

[2] Aitken RJ. The global decline in human fertility: the post-transition trap hypothesis. Life. 2024;14:369. 10.3390/life14030369. 38541694 10.3390/life14030369 PMC10971883

[3] Aitken RJ. The changing tide of human fertility. Hum Reprod. 2022;37:629–38. 10.1093/humrep/deac011. 35079808 10.1093/humrep/deac011 PMC8977063

[4] Mann U, Shiff B, Patel P. Reasons for worldwide decline in male fertility. Curr Opin Urol. 2020;30:296–301. 10.1097/MOU.0000000000000745. 32168194 10.1097/MOU.0000000000000745

[5] Tamburrino L, Traini G, Marcellini A, Vignozzi L, Baldi E, Marchiani S. cryopreservation of human spermatozoa: functional, molecular and clinical aspects. Int J Mol Sci. 2023;24:4656. 10.3390/ijms24054656. 36902084 10.3390/ijms24054656 PMC10002855

[6] Daudin M, Rives N, Walschaerts M, Drouineaud V, Szerman E, Koscinski I, et al. Sperm cryopreservation in adolescents and young adults with cancer: results of the French national sperm banking network (CECOS). Fertil Steril. 2015;103:478-86.e1. 10.1016/j.fertnstert.2014.11.012. 25527232 10.1016/j.fertnstert.2014.11.012

[7] Di Santo M, Tarozzi N, Nadalini M, Borini A. Human sperm cryopreservation: update on techniques, effect on DNA Integrity, and implications for ART. Adv Urol. 2012;2012:854837. 10.1155/2012/854837. 22194740 10.1155/2012/854837 PMC3238352

[8] Hammadeh ME, Askari AS, Georg T, Rosenbaum P, Schmidt W. Effect of freeze-thawing procedure on chromatin stability, morphological alteration and membrane integrity of human spermatozoa in fertile and subfertile men. Int J Androl. 1999;22:155–62. 10.1046/j.1365-2605.1999.00162.x. 10367235 10.1046/j.1365-2605.1999.00162.x

[9] Menzel V, Richter E, Helke C, Bürk BT, Erb HHH, Leike S, et al. Utilization of sperm cryopreservation in patients with testicular cancer. J Cancer Res Clin Oncol. 2024;150:201. 10.1007/s00432-024-05725-2. 38630148 10.1007/s00432-024-05725-2 PMC11024033

[10] Royere D, Barthelemy C, Hamamah S, Lansac J. Cryopreservation of spermatozoa: a 1996 review. Hum Reprod Update. 1996;2:553–9. 10.1093/humupd/2.6.553. 9111188 10.1093/humupd/2.6.553

[11] Bunge RG, Sherman JK. Fertilizing capacity of frozen human spermatozoa. Nature. 1953;172(4382):767–8. 10.1038/172767b0. 13111181 10.1038/172767b0

[12] Hungerford AJ, Bakos HW, Aitken RJ. Addition of vitamin C mitigates the loss of antioxidant capacity, vitality and DNA integrity in cryopreserved human semen samples. Antioxidants. 2024;13:247. 10.3390/antiox13020247. 38397845 10.3390/antiox13020247 PMC10885938

[13] World Health Organisation (WHO) WHO laboratory manual for the examination and processing of human semen. Sixth Edition. Geneva: WHO Press. 2021. https://www.who.int/publications/i/item/9789240030787. Accessed 7 May 2025.

[14] Hungerford A, Bakos HW, Aitken RJ. Sperm cryopreservation: current status and future developments. Reprod Fertil Dev. 2023;35:265–81. 10.1071/RD22219. 36521496 10.1071/RD22219

[15] Fu L, Ma J, Chen L, Guo Y, Li W, Zhang X, Lu W, Wang S, Liu Y. Enhancement of frozen-thawed human sperm quality with zinc as a cryoprotective additive. Med Sci Monit. 2024;30:e942946. 10.12659/MSM.942946. 38698627 10.12659/MSM.942946 PMC11075574

[16] Salehi E, Shadboorestan A, Mohammadi-Bardbori A, Mousavi A, Kargar-Abargouei E, Sarkoohi P, et al. Effect of crocin and quercetin supplementation in cryopreservation medium on post-thaw human sperm quality. Cell Tissue Bank. 2024;25:531–40. 10.1007/s10561-023-10110-3. 37776436 10.1007/s10561-023-10110-3

[17] Najafi L, Halvaei I, Movahedin M. Canthaxanthin protects human sperm parameters during cryopreservation. Andrologia. 2019;51:e13389. 10.1111/and.13389. 31402476 10.1111/and.13389

[18] Cankut S, Dinc T, Cincik M, Ozturk G, Selam B. Evaluation of sperm DNA fragmentation via Halosperm technique and TUNEL assay before and after cryopreservation. Reprod Sci. 2019;26:1575–81. 10.1177/1933719119828096. 30717629 10.1177/1933719119828096

[19] Biggers JD, Whitten WK, Whittingham DG. The culture of mouse embryos in vitro. In: JC Daniel JC (ed.) Methods in mammalian embryology. San Francisco: Freeman. 1971, pp 86–116.

[20] Mantovani R, Rora A, Falomo ME, Bailoni L, Vincenti L. Comparison between glycerol and ethylene glycol for the cryopreservation of equine spermatozoa: semen quality assessment with standard analyses and with the hypoosmotic swelling test. Reprod Nutr Dev. 2002;42:217–26. 10.1051/rnd:2002020. 12405450 10.1051/rnd:2002020

[21] Rota A, Milani C, Cabianca G, Martini M. Comparison between glycerol and ethylene glycol for dog semen cryopreservation. Theriogenology. 2006;65:1848–58. 10.1016/j.theriogenology.2005.10.015. 16310841 10.1016/j.theriogenology.2005.10.015

[22] Keros V, Rosenlund B, Hultenby K, Aghajanova L, Levkov L, Hovatta O. Optimizing cryopreservation of human testicular tissue: comparison of protocols with glycerol, propanediol and dimethylsulphoxide as cryoprotectants. Hum Reprod. 2005;20:1676–87. 10.1093/humrep/deh797. 15860503 10.1093/humrep/deh797

[23] Phillis JW, Estevez AY, O’Regan MH. Protective effects of the free radical scavengers, dimethyl sulfoxide and ethanol, in cerebral ischemia in gerbils. Neurosci Lett. 1998;244:109–11. 10.1016/s0304-3940(98)00139-6. 9572597 10.1016/s0304-3940(98)00139-6

[24] Watson PF, Duncan AE. Effect of salt concentration and unfrozen water fraction on the viability of slowly frozen ram spermatozoa. Cryobiology. 1988;25:131–42. 10.1016/0011-2240(88)90006-5. 3371058 10.1016/0011-2240(88)90006-5

[25] Câmara DR, Kastelic JP, Thundathil JC. Role of the Na^+^/K^+^-ATPase ion pump in male reproduction and embryo development. Reprod Fertil Dev. 2017;29:1457–67. 10.1071/RD16091. 27456939 10.1071/RD16091

[26] Aitken RJ, Finnie JM, Muscio L, Whiting S, Connaughton HS, Kuczera L, et al. Potential importance of transition metals in the induction of DNA damage by sperm preparation media. Hum Reprod. 2014;29:2136–47. 10.1093/humrep/deu204. 25141857 10.1093/humrep/deu204

[27] Hungerford AJ, Bakos HW, Aitken RJ. Analysis of sperm separation protocols for isolating cryopreserved human spermatozoa. Reprod Fertil. 2023;4:e220133. 10.1530/RAF-22-0133. 37000632 10.1530/RAF-22-0133 PMC10160538

[28] Bromfield EG, Aitken RJ, Gibb Z, Lambourne SR, Nixon B. Capacitation in the presence of methyl-β-cyclodextrin results in enhanced zona pellucida-binding ability of stallion spermatozoa. Reproduction. 2013;147:153–66. 10.1530/REP-13-0393. 24194571 10.1530/REP-13-0393

[29] Hong CY, Chiang BN, Turner P. Calcium ion is the key regulator of human sperm function. Lancet. 1984;2(8417–8418):1449–51. 10.1016/s0140-6736(84)91634-9. 6151055 10.1016/s0140-6736(84)91634-9

[30] Fu L, Ma J, Chen L, Guo Y, Li W, Zhang X, et al. Enhancement of frozen-thawed human sperm quality with zinc as a cryoprotective additive. Med Sci Monit. 2024;30:e9429463. 10.12659/MSM.942946. 10.12659/MSM.942946 PMC11075574 38698627

[31] Najafi A, Kia HD, Mohammadi H, Najafi MH, Zanganeh Z, Sharafi M, et al. Different concentrations of cysteamine and ergothioneine improve microscopic and oxidative parameters in ram semen frozen with a soybean lecithin extender. Cryobiology. 2014;69:68–73. 10.1016/j.cryobiol.2014.05.004. 24854868 10.1016/j.cryobiol.2014.05.004

[32] Camp JE, Nyamini SB, Scott FJ. Cyrene™ is a green alternative to DMSO as a solvent for antibacterial drug discovery against ESKAPE pathogens. RSC Med Chem. 2019;11:111–7. 10.1039/c9md00341j. 33479610 10.1039/c9md00341j PMC7522793

[33] Pilikian S, Mimouni P. Comparative study of the kinetics of the acrosome reaction and survival of human spermatozoa in various media. Int J Androl. 1988;11:465–72. 10.1111/j.1365-2605.1988.tb01020.x. 3215701 10.1111/j.1365-2605.1988.tb01020.x

[34] Cavarocchi E, Whitfield M, Chargui A, Stouvenel L, Lorès P, Coutton C, et al. The sodium/proton exchanger SLC9C1 (sNHE) is essential for human sperm motility and fertility. Clin Genet. 2021;99:684–93. 10.1111/cge.13927. 33462806 10.1111/cge.13927

[35] Torres-Flores V, Picazo-Juárez G, Hernández-Rueda Y, Darszon A, González-Martínez MT. Sodium influx induced by external calcium chelation decreases human sperm motility. Hum Reprod. 2011;26:2626–35. 10.1093/humrep/der237. 21810864 10.1093/humrep/der237 PMC3174032

[36] Song Q, Guo R, Wei W, Lv L, Song Z, Feng R, et al. Histidine-alleviated hepatocellular death in response to 4-hydroxynonenal contributes to the protection against high-fat diet-induced liver injury. J Funct Foods. 2017;39:74–83.

[37] Moazamian R, Polhemus A, Connaughton H, Fraser B, Whiting S, Gharagozloo P, et al. Oxidative stress and human spermatozoa: diagnostic and functional significance of aldehydes generated as a result of lipid peroxidation. Mol Hum Reprod. 2015;21:502–15. 10.1093/molehr/gav014. 25837702 10.1093/molehr/gav014

[38] Zhang L, Wang Y, Sohail T, Kang Y, Niu H, Sun X, et al. Effects of taurine on sperm quality during room temperature storage in Hu sheep. Animals. 2021;11:2725. 10.3390/ani11092725. 34573691 10.3390/ani11092725 PMC8470579

[39] Ijaz A, Ducharme R. Effect of various extenders and taurine on survival of stallion sperm cooled to 5 degrees C. Theriogenology. 1995;44:1039–50. 10.1016/0093-691x(95)00290-o. 16727798 10.1016/0093-691x(95)00290-o

[40] Chan SY. Taurine and human spermatozoal capacitation. Cell Biol Int Rep. 1985;9:127–30. 10.1016/0309-1651(85)90086-4. 3978719 10.1016/0309-1651(85)90086-4

[41] Yun JI, Gong SP, Song YH, Lee ST. Effects of combined antioxidant supplementation on human sperm motility and morphology during sperm manipulation in vitro. Fertil Steril. 2013;100:373–8. 10.1016/j.fertnstert.2013.04.01. 23651626 10.1016/j.fertnstert.2013.04.015

[42] Hamamah S, Gatti JL. Role of the ionic environment and internal pH on sperm activity. Hum Reprod. 1998;13(Suppl 4):20–30. 10.1093/humrep/13.suppl_4.20. 10091055 10.1093/humrep/13.suppl_4.20

[43] Hammitt DG, Bedia E, Rogers PR, Syrop CH, Donovan JF, Williamson RA. Comparison of motility stimulants for cryopreserved human semen. Fertil Steril. 1989;52:495–502. 10.1016/s0015-0282(16)60925-1. 2550282 10.1016/s0015-0282(16)60925-1

[44] Hadi Z, Ahmadi E, Shams-Esfandabadi N, Davoodian N, Shirazi A, Moradian M. Polyvinyl alcohol addition to freezing extender can improve the post-thaw quality, longevity and in vitro fertility of ram epididymal spermatozoa. Cryobiology. 2024;114:104853. 10.1016/j.cryobiol.2024.104853. 38301951 10.1016/j.cryobiol.2024.104853

[45] Wu Z, Zheng X, Luo Y, Huo F, Dong H, Zhang G, et al. Cryopreservation of stallion spermatozoa using different cryoprotectants and combinations of cryoprotectants. Anim Reprod Sci. 2015;163:75–81. 10.1016/j.anireprosci.2015.09.020. 26573763 10.1016/j.anireprosci.2015.09.020

[46] Domingo P, Olaciregui M, González N, De Blas I, Gil L. Long-term preservation of freeze-dried rabbit sperm by adding rosmarinic acid and different chelating agents. Cryobiology. 2018;81:174–7. 10.1016/j.cryobiol.2018.01.004. 29366763 10.1016/j.cryobiol.2018.01.004

[47] Olaciregui M, Luño V, González N, Domingo P, de Blas I, Gil L. Chelating agents in combination with rosmarinic acid for boar sperm freeze-drying. Reprod Biol. 2017;17:193–8. 10.1016/j.repbio.2017.05.001. 28576621 10.1016/j.repbio.2017.05.001

[48] Hussain N, Andrabi SMH, Mehmood MU. Effect of EDTA as chelating agent in extender on the post thaw quality of buffalo bull spermatozoa. Cryo Letters. 2019;40:159–63. 31095664

[49] Holt WV. Basic aspects of frozen storage of semen. Anim Reprod Sci. 2000;62:3–22. 10.1016/s0378-4320(00)00152-4. 10924818 10.1016/s0378-4320(00)00152-4

[50] Kadirvel G, Kumar S, Kumaresan A, Kathiravan P. Capacitation status of fresh and frozen-thawed buffalo spermatozoa in relation to cholesterol level, membrane fluidity and intracellular calcium. Anim Reprod Sci. 2009;116:244–53. 10.1016/j.anireprosci.2009.02.003. 19261396 10.1016/j.anireprosci.2009.02.003

[51] Williams KM, Ford WC. Effects of Ca-ATPase inhibitors on the intracellular calcium activity and motility of human spermatozoa. Int J Androl. 2003;26:366–75. 10.1111/j.1365-2605.2003.00438.x. 14636222 10.1111/j.1365-2605.2003.00438.x

[52] Rhemrev JP, van Overveld FW, Haenen GR, Teerlink T, Bast A, Vermeiden JP. Quantification of the nonenzymatic fast and slow TRAP in a postaddition assay in human seminal plasma and the antioxidant contributions of various seminal compounds. J Androl. 2000;21:913–20. 11105918

[53] Sadeghi N, Boissonneault G, Tavalaee M, Nasr-Esfahani MH. Oxidative versus reductive stress: a delicate balance for sperm integrity. Syst Biol Reprod Med. 2023;69:20–31. 10.1080/19396368.2022.2119181. 36215401 10.1080/19396368.2022.2119181

[54] Abdolsamadi M, Mohammadi F, Nashtaei MS, Teimouri M, Sardar R, Dayani M, et al. Does myoinositol supplement improve sperm parameters and DNA integrity in patients with oligoasthenoteratozoospermia after the freezing-thawing process? Cell Tissue Bank. 2020;21:99–106. 10.1007/s10561-019-09801-7. 31845062 10.1007/s10561-019-09801-7

[55] Azizi M, Cheraghi E, Soleimani MM. Effect of myo-inositol on sperm quality and biochemical factors in cryopreserved semen of patients with asthenospermia. Andrologia. 2022;54:e14528. 10.1111/and.14528. 35841196 10.1111/and.14528

[56] Jawad A, Oh D, Choi H, Kim M, Cai L, Lee J, et al. Myo-inositol improves the viability of boar sperm during liquid storage. Front Vet Sci. 2023;10:1150984. 10.3389/fvets.2023.1150984. 37565079 10.3389/fvets.2023.1150984 PMC10411888

[57] Koppers AJ, Mitchell LA, Wang P, Lin M, Aitken RJ. Phosphoinositide 3-kinase signalling pathway involvement in a truncated apoptotic cascade associated with motility loss and oxidative DNA damage in human spermatozoa. Biochem J. 2011;436:687–98. 10.1042/BJ20110114. 21470189 10.1042/BJ20110114

[58] Hallam J, Burton P, Sanders K. Poor sperm chromatin condensation is associated with cryopreservation-induced DNA fragmentation and cell death in human spermatozoa. J Clin Med. 2024. 10.3390/jcm13144156. 39064196 10.3390/jcm13144156 PMC11277714

[59] Raad G, Lteif L, Lahoud R, Azoury J, Azoury J, Tanios J, et al. Cryopreservation media differentially affect sperm motility, morphology and DNA integrity. Andrology. 2018;6:836–45. 10.1111/andr.12531. 30105872 10.1111/andr.12531

