# Supplementary material for: Development of an improved medium for the preservation of human spermatozoa
Source: J Assist Reprod Genet. 2025 May 30;42(7):2167–80. doi: 10.1007/s10815-025-03525-2 (PMC12356804; doi:10.1007/s10815-025-03525-2)
Supplement: Supplementary file 1 — Supplementary file1 (PPTX 109 KB) [file 10815_2025_3525_MOESM1_ESM.pptx]

## Slide 1
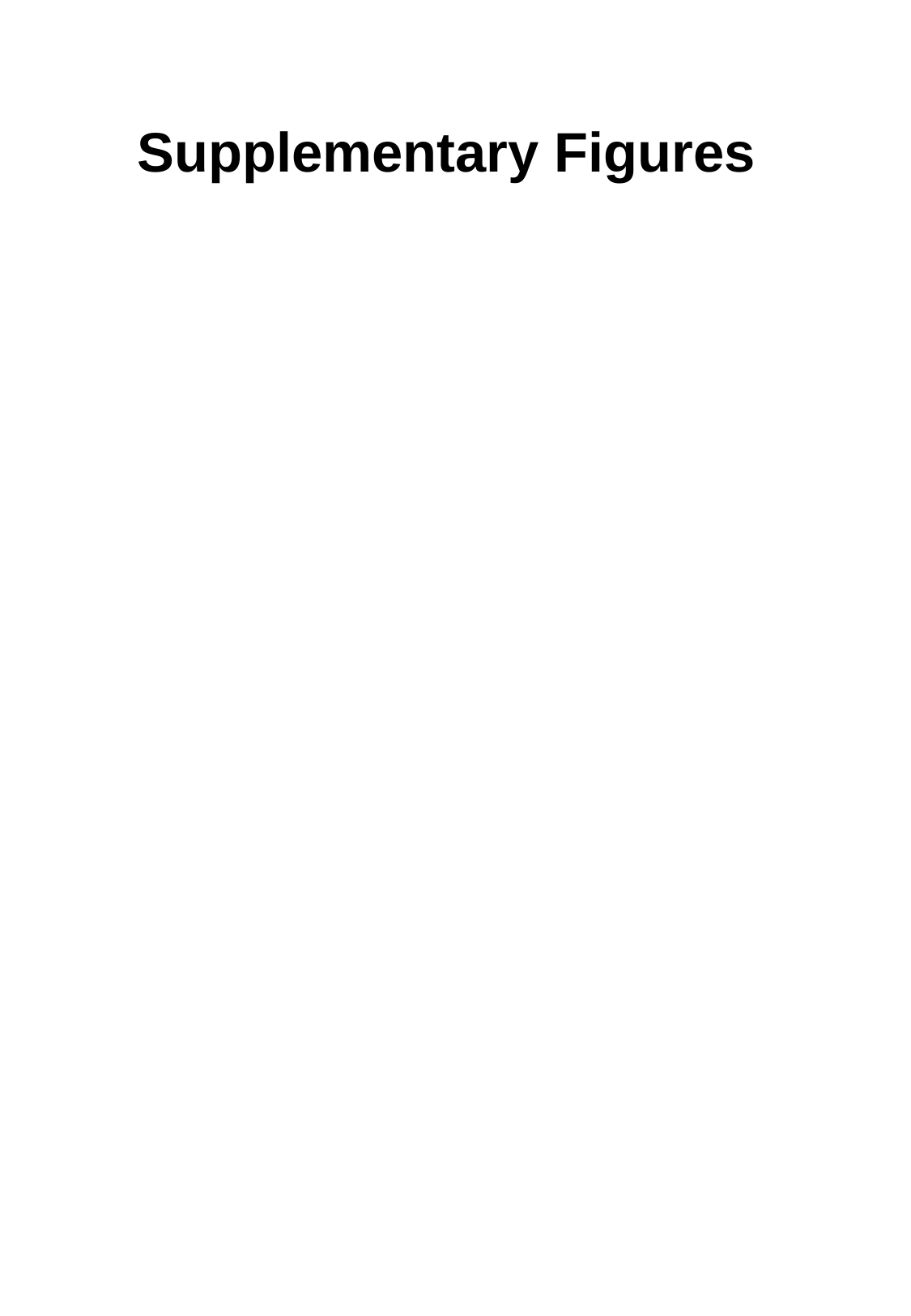

# Supplementary Figures

## Slide 2
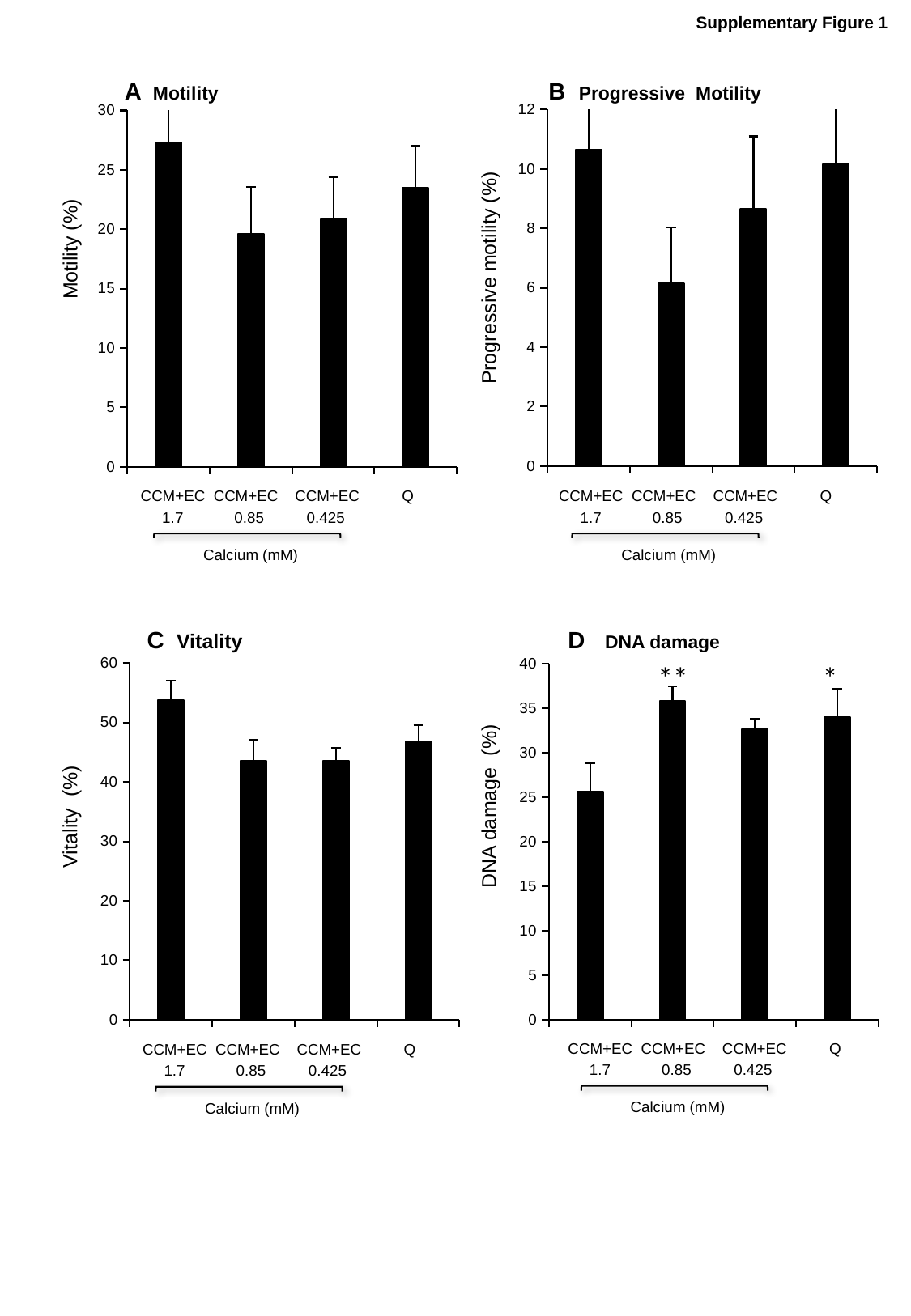

Supplementary Figure 1
A Motility
B Progressive Motility
### Chart
| Category | |
|---|---|
| S1EC | 10.64 |
| S1EC 1/2 CaCl2 | 6.14 |
| S1EC 1/4 CaCl2 | 8.66 |
| Quinns | 10.16 |
### Chart
| Category | |
|---|---|
| S1EC | 27.32 |
| S1EC 1/2 CaCl2 | 19.64 |
| S1EC 1/4 CaCl2 | 20.9 |
| Quinns | 23.52 |Motility (%)
Progressive motility (%)
CCM+EC CCM+EC CCM+EC Q
1.7 0.85 0.425
Calcium (mM)
CCM+EC CCM+EC CCM+EC Q
1.7 0.85 0.425
Calcium (mM)
C Vitality
D DNA damage
### Chart
| Category | |
|---|---|
| S1EC | 53.8 |
| S1EC 1/2 CaCl2 | 43.6 |
| S1EC 1/4 CaCl2 | 43.6 |
| Quinns | 46.8 |
### Chart
| Category | |
|---|---|
| S1EC | 25.6 |
| S1EC 1/2 CaCl2 | 35.8 |
| S1EC 1/4 CaCl2 | 32.6 |
| Quinns | 34.0 |*
**
DNA damage (%)
Vitality (%)
CCM+EC CCM+EC CCM+EC Q
1.7 0.85 0.425
Calcium (mM)
CCM+EC CCM+EC CCM+EC Q
1.7 0.85 0.425
Calcium (mM)

## Slide 3
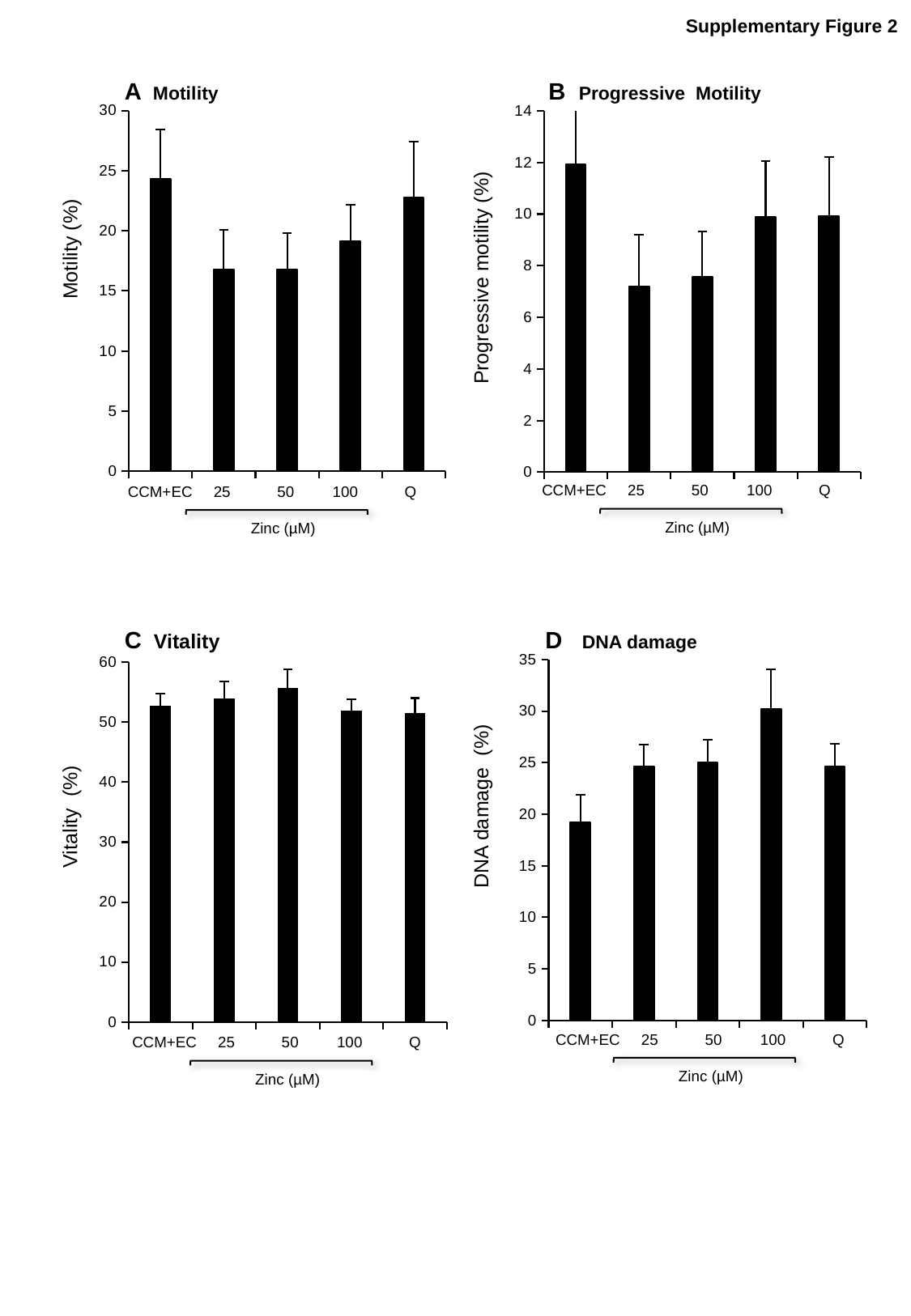

Supplementary Figure 2
### Chart
| Category | |
|---|---|
| S1EC | 11.92 |
| S1EC+ZnSO4 25uM | 7.2 |
| S1EC+ZnSO4 50uM | 7.56 |
| S1EC+ZnSO4 100uM | 9.88 |
| Quinns | 9.92 |
### Chart
| Category | |
|---|---|
| S1EC | 24.34 |
| S1EC+ZnSO4 25uM | 16.8 |
| S1EC+ZnSO4 50uM | 16.8 |
| S1EC+ZnSO4 100uM | 19.16 |
| Quinns | 22.76 |A Motility
B Progressive Motility
Motility (%)
Progressive motility (%)
CCM+EC 25 50 100 Q
Zinc (µM)
CCM+EC 25 50 100 Q
Zinc (µM)
### Chart
| Category | |
|---|---|
| S1EC | 52.6 |
| S1EC+ZnSO4 25uM | 53.8 |
| S1EC+ZnSO4 50uM | 55.6 |
| S1EC+ZnSO4 100uM | 51.8 |
| Quinns | 51.4 |C Vitality
D DNA damage
### Chart
| Category | |
|---|---|
| S1EC | 19.2 |
| S1EC+ZnSO4 25uM | 24.6 |
| S1EC+ZnSO4 50uM | 25.0 |
| S1EC+ZnSO4 100uM | 30.2 |
| Quinns | 24.6 |DNA damage (%)
Vitality (%)
CCM+EC 25 50 100 Q
Zinc (µM)
CCM+EC 25 50 100 Q
Zinc (µM)

## Slide 4
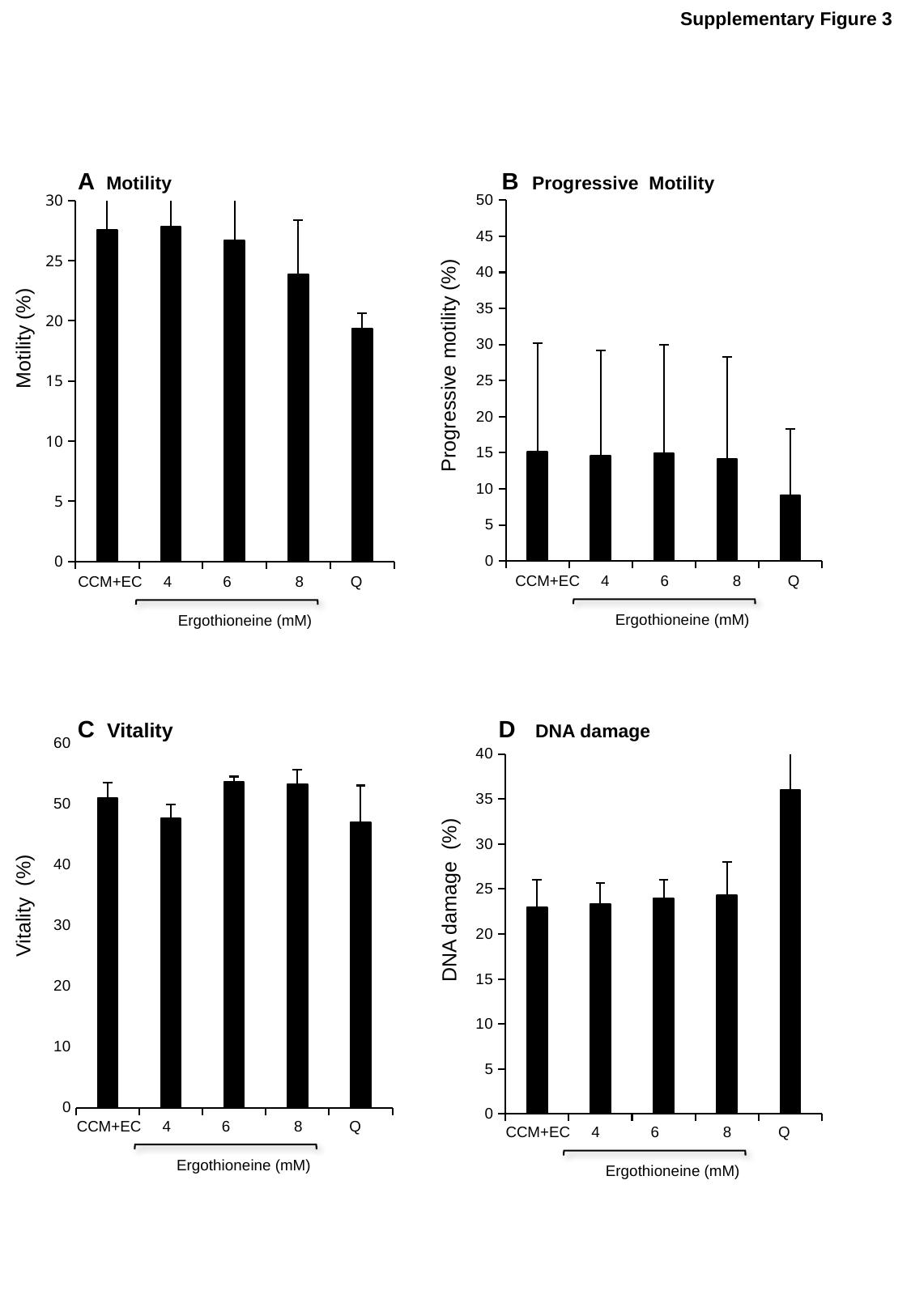

Supplementary Figure 3
### Chart
| Category | |
|---|---|
| S1EC | 27.533333 |
| S1EC+ERT 4mM | 27.8 |
| S1EC+ERT 6mM | 26.666667 |
| S1EC+ERT 8mM | 23.866667 |
| Quinns | 19.366667 |A Motility
B Progressive Motility
### Chart
| Category | |
|---|---|
| S1EC | 15.1 |
| S1EC+ERT 4mM | 14.566667 |
| S1EC+ERT 6mM | 14.966667 |
| S1EC+ERT 8mM | 14.133333 |
| Quinns | 9.1333333 |Motility (%)
Progressive motility (%)
CCM+EC 4 6 8 Q
Ergothioneine (mM)
CCM+EC 4 6 8 Q
Ergothioneine (mM)
### Chart
| Category | |
|---|---|
| S1EC | 23.0 |
| S1EC+ERT 4mM | 23.333333 |
| S1EC+ERT 6mM | 24.0 |
| S1EC+ERT 8mM | 24.333333 |
| Quinns | 36.0 |
### Chart
| Category | |
|---|---|
| S1EC | 51.0 |
| S1EC+ERT 4mM | 47.666667 |
| S1EC+ERT 6mM | 53.666667 |
| S1EC+ERT 8mM | 53.333333 |
| Quinns | 47.0 |C Vitality
D DNA damage
DNA damage (%)
Vitality (%)
CCM+EC 4 6 8 Q
Ergothioneine (mM)
CCM+EC 4 6 8 Q
Ergothioneine (mM)

## Slide 5
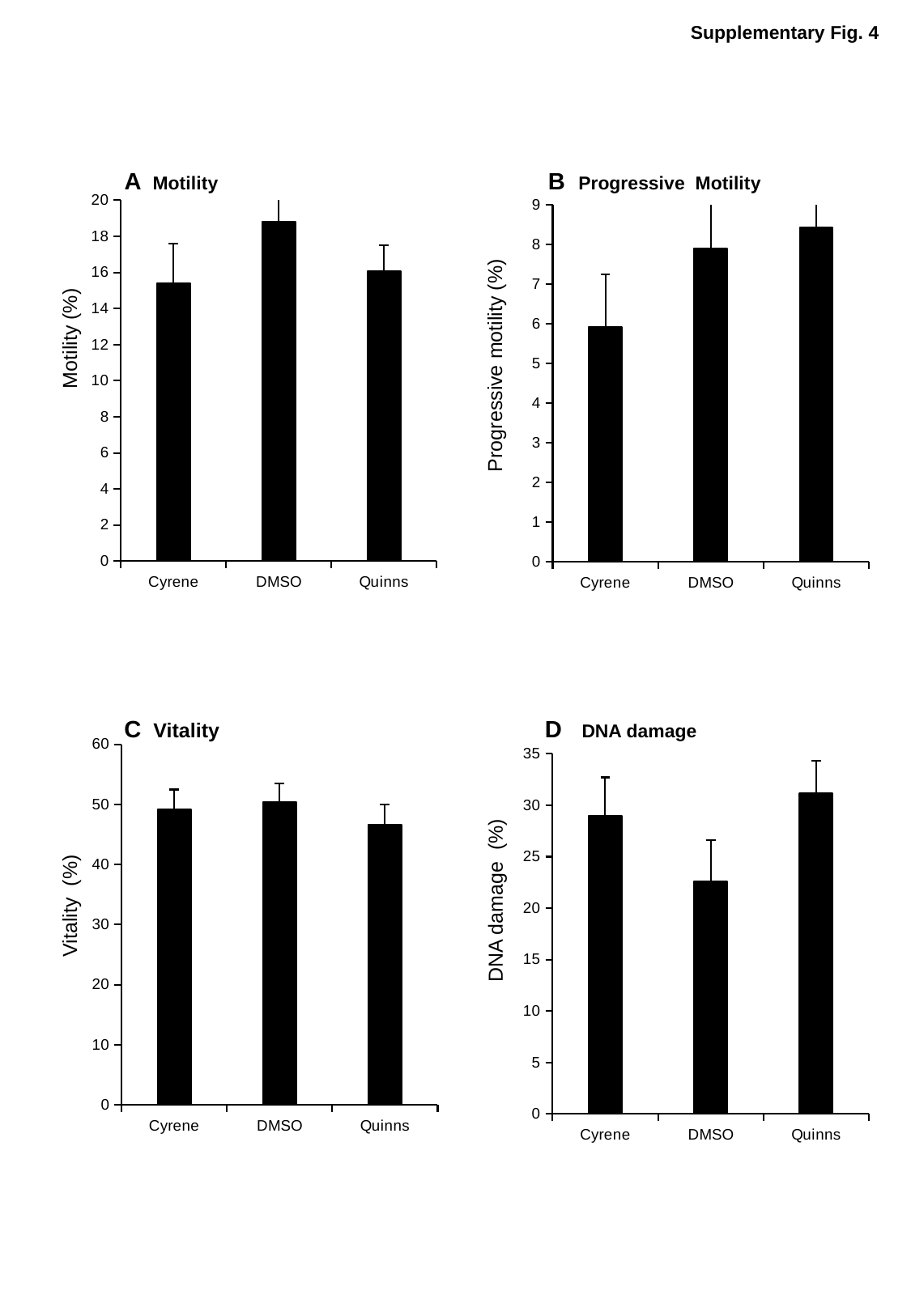

Supplementary Fig. 4
### Chart
| Category | |
|---|---|
| Cyrene | 5.92 |
| DMSO | 7.9 |
| Quinns | 8.44 |
### Chart
| Category | |
|---|---|
| Cyrene | 15.38 |
| DMSO | 18.82 |
| Quinns | 16.06 |A Motility
B Progressive Motility
Motility (%)
Progressive motility (%)
### Chart
| Category | |
|---|---|
| Cyrene | 49.2 |
| DMSO | 50.4 |
| Quinns | 46.6 |
### Chart
| Category | |
|---|---|
| Cyrene | 29.0 |
| DMSO | 22.6 |
| Quinns | 31.2 |C Vitality
D DNA damage
DNA damage (%)
Vitality (%)
